# Supplementary material for: GOLT1B Activation in Hepatitis C Virus-Infected Hepatocytes Links ER Trafficking and Viral Replication
Source: Pathogens. 2021 Dec 31;11(1):46. doi: 10.3390/pathogens11010046 (PMC8781247; doi:10.3390/pathogens11010046)
Supplement: Supplementary file 1 [file pathogens-11-00046-s001.zip › pathogens-1494910-supplementary.pdf]

Supplementary figure S1

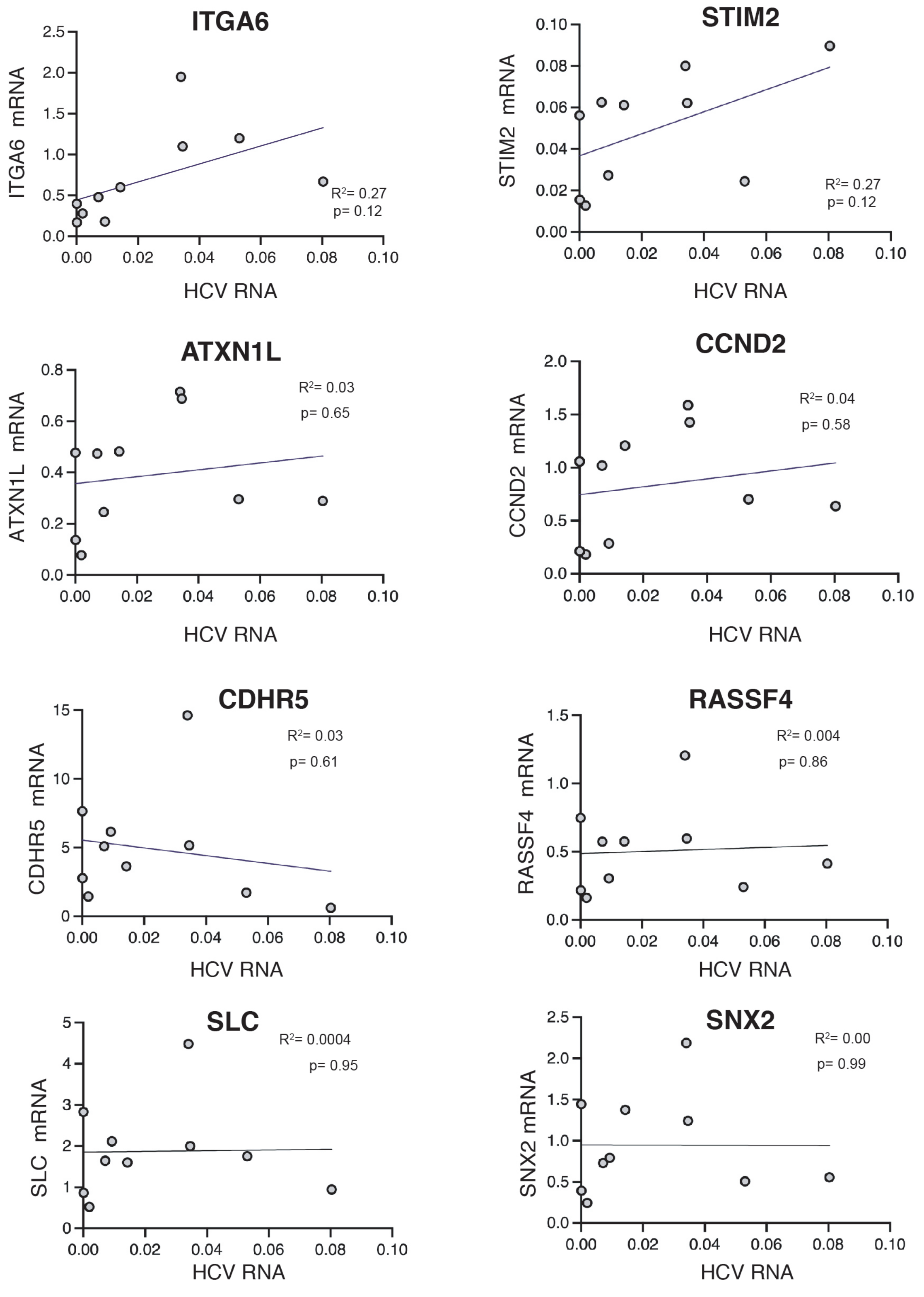

Figure S1: Correlations of HCV RNA levels and eight target genes in non-tumoral regions of HCC patients. mRNA levels of the 8 indicated genes were quantified by qPCR in non tumoral and tumoral samples from ten patients with HCC of HCV etiology. The two genes with positive correlations (CLOCK and GOLT1B) are shown in Figure 4A. The R-squared and p-values from Pearson correlation tests (two-tailed) are indicated.

Supplementary figure S2

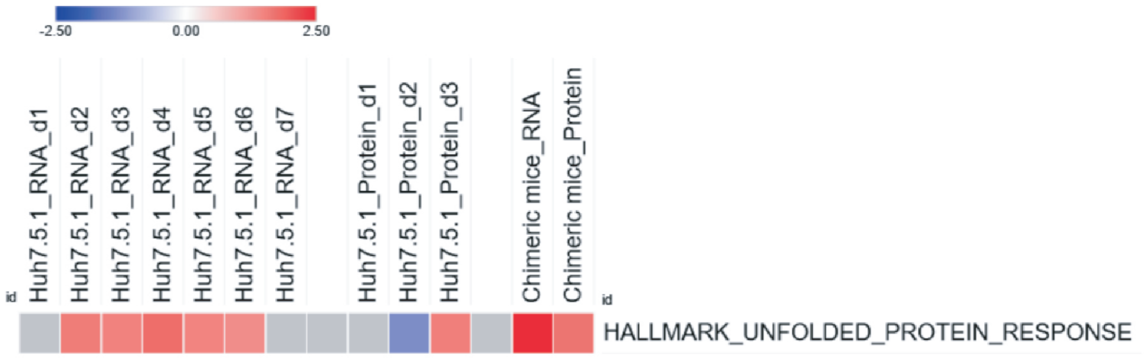

Figure S2: HCV infection induces an unfolded protein response in Huh7.5.1 and human liver chimeric mice. Gene set enrichment analysis (GSEA) of RNA-seq and proteomic data of HCV-infection time course of quiescent Huh7.5.1 (Huh7.5.1dif) relative to mock-infected cells (n=2) and from HCV-infected human liver chimeric mice from [10]. Significant changes of the normalized enrichment scores (NES) are indicated in color. The statistical cutoff for GSEA of liver tissues was a false discovery rate of Q<0.05 and for Huh7.5.1dif was P<0.005. Enrichment (upregulation)=red, negative enrichment (downregulation)=blue. The Hallmark gene set have been obtained from the Molecular Signature Database [38].

Supplementary Table S1

3' sequence complementary to the secondary detection probe (FLAP-Cy3) : TTACACTCGGACCTCGTCGACATGCATT
FLAP-Y Cy3: /5Cy3/AA TGC ATG TCG ACG AGG TCC GAG TGT AA/3Cy3Sp/

Sequence Name	Sequence
Y-HCV-1b-1	GGCACGGGGAGACAGGCTGTGATATA**TTACACTCGGACCTCGTCGACATGCATT**
Y-HCV-1b-2	GGGACAGTAGCTTAGCGCGGACACTTTTACACTCGGACCTCGTCGACATGCATT
Y-HCV-1b-3	ATGTCTCCAGACTCGCAAGGGTGGTATTACACTCGGACCTCGTCGACATGCATT
Y-HCV-1b-4	CCAAGTTTCCTGAGGCATGAAGCCACTTACACTCGGACCTCGTCGACATGCATT
Y-HCV-1b-5	GATCTGAGGTAGGTCAAGTGGCTCAATGTTACACTCGGACCTCGTCGACATGCATT
Y-HCV-1b-6	CCCATAAGGTGGGCGCATACATGATGTTACACTCGGACCTCGTCGACATGCATT
Y-HCV-1b-7	GCCGCAGAGGCCTTCAAGTAACATGTTTACACTCGGACCTCGTCGACATGCATT
Y-HCV-1b-8	GGTATTACCGCAGCTAGTCGTCAGCATTACACTCGGACCTCGTCGACATGCATT
Y-HCV-1b-9	CGATAACCGCAGTTCTGCCCTTTTGAATTACACTCGGACCTCGTCGACATGCATT
Y-HCV-1b-10	CCGATATAAAGCCGCTCTGTGAGCGATTACACTCGGACCTCGTCGACATGCATT
Y-HCV-1b-11	CTTCGGGGGCCAAGTCACAACATTGGTTACACTCGGACCTCGTCGACATGCATT
Y-HCV-1b-12	TTGATTTCCAGGCATTCACCAGGAACTTTACACTCGGACCTCGTCGACATGCATT
Y-HCV-1b-13	CCGCTGTCCAGGAGAGTACTGGAATCTTACACTCGGACCTCGTCGACATGCATT
Y-HCV-1b-14	ACGTCGTAAAGGGCCATTTTCTCGCACTTACACTCGGACCTCGTCGACATGCATT
Y-HCV-1b-15	TGTGGTGACGCAGCAAAGAGTTGCTCATTACACTCGGACCTCGTCGACATGCATT
Y-HCV-1b-16	TGGGAGCTGTGACCCAACCAGGTATTTTACACTCGGACCTCGTCGACATGCATT
Y-HCV-1b-17	GAATGTGACCTCCTCCCGTAGGAGAGTTACACTCGGACCTCGTCGACATGCATT
Y-HCV-1b-18	CCAACGATCCTCATGGAACCGTTTTTGATTACACTCGGACCTCGTCGACATGCATT
Y-HCV-1b-19	TCAGTCAACACCGTGCATATCCAGTCTTACACTCGGACCTCGTCGACATGCATT
Y-HCV-1b-20	GCATGGCGTGGAGCAGTCCTCATTAATTACACTCGGACCTCGTCGACATGCATT
Y-HCV-1b-21	GTGGAGCCTCTTCAACAGCTGAGTGATTACACTCGGACCTCGTCGACATGCATT
Y-HCV-1b-22	CGAAGCGAACGCTATCAGCCGGTTCATTACACTCGGACCTCGTCGACATGCATT
Y-HCV-1b-23	CGCTCATGACCTTAAAGGCCACGAGTTTACACTCGGACCTCGTCGACATGCATT
Y-HCV-1b-24	TAGAGGCTGTGAATGCCATCAGTGATGTTACACTCGGACCTCGTCGACATGCATT
Y-HCV-1b-25	TGCTAAGTACTGTATCCCGCTGATGAATTACACTCGGACCTCGTCGACATGCATT
Y-HCV-1b-26	CATGTGCTTCGCCCAGAAGGTCTCAATTACACTCGGACCTCGTCGACATGCATT
Y-HCV-1b-27	TGTTGTCAGGCAATACGCGGCCAGAGTTACACTCGGACCTCGTCGACATGCATT
Y-HCV-1b-28	GCCGACATGCATGCCATGATGTATTTTTACACTCGGACCTCGTCGACATGCATT
Y-HCV-1b-29	ACACTTCCACATTTGATCCCACGATGTTACACTCGGACCTCGTCGACATGCATT
Y-HCV-1b-30	GCAGACGGGCAACCCTGGTGTATTTATTACACTCGGACCTCGTCGACATGCATT
Y-HCV-1b-31	GAGGGCCGTTCTCCTGGAGTCACAAATTACACTCGGACCTCGTCGACATGCATT
Y-HCV-1b-32	TAGGGTCCAAGCTGAAATCGACTGTCTTTACACTCGGACCTCGTCGACATGCATT
Y-HCV-1b-33	CGCTGGTCGGTATGACGGACACATCATTACACTCGGACCTCGTCGACATGCATT
Y-HCV-1b-34	CCATAGAAGGGGATCTCTCCAGTGTTGTTACACTCGGACCTCGTCGACATGCATT
Y-HCV-1b-35	TGCCGATGCCCAAGATGGTAGTCGAGTTACACTCGGACCTCGTCGACATGCATT
Y-HCV-1b-36	TAGGGTCGACACCATGTGCCTTAGACTTACACTCGGACCTCGTCGACATGCATT
Y-HCV-1b-37	CGGGGACCGCATAGTAGTTTCCATAGATTACACTCGGACCTCGTCGACATGCATT
Y-HCV-1b-38	CAACGGGTACAAAGTCCACCGCCTTCTTACACTCGGACCTCGTCGACATGCATT
Y-HCV-1b-39	GTTGACGCAGGTCGCCAGGAAAGATTGTTTACACTCGGACCTCGTCGACATGCATT
Y-HCV-1b-40	CTGTGAGGCTAGTGATGATGCAGCCATTACACTCGGACCTCGTCGACATGCATT
Y-HCV-1b-41	TTCAAGACTATCGGCCGGTCCCAGAATTACACTCGGACCTCGTCGACATGCATT
Y-HCV-1b-42	TAAACGTACGTACCTGTCAGTGCGGCTTACACTCGGACCTCGTCGACATGCATT
Y-HCV-1b-43	CGCGCACGAAGTACGGCACTCTAGTTTTACACTCGGACCTCGTCGACATGCATT
Y-HCV-1b-44	CGCATTGAGGACCACCAGGTTCTCTATTACACTCGGACCTCGTCGACATGCATT
Y-HCV-1b-45	CGCTGACCCTATACCGTACAGGTATTTTACACTCGGACCTCGTCGACATGCATT
Y-HCV-1b-46	ACGTCCACGATGTTCTGATGGAGATGGTTACACTCGGACCTCGTCGACATGCATT
Y-HCV-1b-47	TGTTGTAGACAGCAGCAGCGGGCTAATTACACTCGGACCTCGTCGACATGCATT
Y-HCV-1b-48	GTAAAGTTGACAGTGCAGGGGTAGTGCCATTACACTCGGACCTCGTCGACATGCATT
Y-HCV-1b-49	CTGTATGGGTAGTCAACCATGCACCTTTACACTCGGACCTCGTCGACATGCATT
Y-HCV-1b-50	CATGTACAGCCGAACCAGTTGCCTTGTTACACTCGGACCTCGTCGACATGCATT
Y-HCV-1b-51	TGAGCGAACTTGTCAATGGGGCGGCATTACACTCGGACCTCGTCGACATGCATT
Y-HCV-1b-52	ACCCAGTGTGGAGGGAGTCATTGCAGTTACACTCGGACCTCGTCGACATGCATT
Y-HCV-1b-53	CACGACAGCTTGTGGGATCCGGAGTAATTACACTCGGACCTCGTCGACATGCATT
Y-HCV-1b-54	GTGACCTGATACGTGGCCGGGATAGATTACACTCGGACCTCGTCGACATGCATT
Y-HCV-1b-55	GCTGCCTCATACACAATGCTTGAGTTTTACACTCGGACCTCGTCGACATGCATT
Y-HCV-1b-56	AGCAGTCGTTCGTGACATGGTACACCTTACACTCGGACCTCGTCGACATGCATT
Y-HCV-1b-57	GGACACGTTGCGCACTTCATAAGCGGTTACACTCGGACCTCGTCGACATGCATT
Y-HCV-1b-58	GCGAAGCCGCATGTGAGGGTATCGATTTACACTCGGACCTCGTCGACATGCATT
Y-HCV-1b-59	TCATTGCCATAGAGGGGCCAAGGGTATTACACTCGGACCTCGTCGACATGCATT
Y-HCV-1b-60	CTATCAGGCAGTACCACAAGGCCGTTTTACACTCGGACCTCGTCGACATGCATT
Y-HCV-1b-61	CAAATCTCCAGGCATTGAGCGGGGTTTTACACTCGGACCTCGTCGACATGCATT

Supplementary Table S2

Up-regulated genes in HCV positive tumor cells

n=98 p-value <0.05 LogFoldChange >2

Gene id	baseMeanA	baseMeanB	foldChange	og2FoldChang	pval
STPG1	0	109	Inf	Inf	0.041
CCDC28B	0	111	Inf	Inf	0.036
SPAG17	0	133	Inf	Inf	0.021
ZNF687-AS1	0	145	Inf	Inf	0.012
RAD51AP2	0	132	Inf	Inf	0.024
STXBP5-AS1	0	147	Inf	Inf	0.011
EXTL3	0	307	Inf	Inf	0.005
LOC392232	0	184	Inf	Inf	0.006
FAM238B	0	118	Inf	Inf	0.036
ZFPL1	0	110	Inf	Inf	0.037
FAM230C	0	140	Inf	Inf	0.017
LINC01055	0	283	Inf	Inf	0.031
GPR137C	0	148	Inf	Inf	0.037
RRN3P2	0	298	Inf	Inf	0
MYH13	0	208	Inf	Inf	0.002
ENDOV	0	306	Inf	Inf	0.029
EFCAB6	0	317	Inf	Inf	0.014
LINC00967	2	179	97.25	6.6	0.03
SPACA9	40	393	9.94	3.31	0.018
GEN1	49	478	9.73	3.28	0.036
KLHL25	3	212	84.01	6.39	0.033
TLR1	4	333	81.54	6.35	0.045
RMI1	42	361	8.51	3.09	0.032
SHCBP1	27	221	8.3	3.05	0.049
OFD1	69	569	8.29	3.05	0.015
ZNF337	3	215	78.43	6.29	0.036
PLCL1	1	398	727.15	9.51	0.005
LINC01359	0	269	701.37	9.45	0.029
ADIPOR2	88	657	7.48	2.9	0.016
HEG1	77	553	7.21	2.85	0.023
MGAT4C	193	1368	7.08	2.82	0.018
LRRC45	5	340	69.5	6.12	0.005
VSX1	3	199	62.7	5.97	0.024
ZNF562	164	1147	6.99	2.81	0
OPHN1	131	842	6.41	2.68	0
DNAJC18	73	448	6.14	2.62	0.041
SUZ12	141	857	6.07	2.6	0.02
ZNF202	4	205	57.59	5.85	0.03
IL17RD	6	331	56.48	5.82	0.002
CYTH3	4	239	53.85	5.75	0.019
LINC00368	67	396	5.92	2.57	0.012
PPARG	83	469	5.62	2.49	0.041
ARSD	178	985	5.54	2.47	0.021
GGT6	67	353	5.28	2.4	0.027
PLA2G2A	270	1386	5.14	2.36	0.011
THRIL	1	268	486.91	8.93	0.007
SIPA1	7	326	45.79	5.52	0.033
MSANTD4	5	239	44.48	5.47	0.049
STK33	0	142	433.31	8.76	0.039
ZBTB3	6	259	43.48	5.44	0.029
AFAP1-AS1	0	163	422.31	8.72	0.017
CCT6B	8	326	41.96	5.39	0.007
UNC119B	7	273	41.87	5.39	0.02
RHNO1	9	373	40.94	5.36	0.008
DNPEP	119	583	4.9	2.29	0.012

GRPR	174	829	4.75	2.25	0.044
KANSL1L	238	1112	4.68	2.23	0.047
PLEKHJ1	69	318	4.59	2.2	0.047
ZNF37A	202	846	4.18	2.06	0.019
NUP88	189	791	4.18	2.06	0.042
XPNPEP1	18	719	39.43	5.3	0.001
CDC42EP1	0	159	358.47	8.49	0.02
ZSCAN5A	0	156	350.55	8.45	0.027
IL12RB2	9	307	35.05	5.13	0.017
DMTN	0	153	349.6	8.45	0.032
NEK5	9	325	34.28	5.1	0.021
BCAS1	8	279	33.27	5.06	0.03
ORC1	9	302	32.99	5.04	0.043
ZFP82	11	355	31.51	4.98	0.029
NISCH	19	597	30.89	4.95	0.01
TBC1D27P	1	228	287.32	8.17	0.02
TEX14	1	228	286.65	8.16	0.002
LOC105372582	1	393	281.24	8.14	0.005
THUMPD2	1	345	262.09	8.03	0.016
TMEM189	1	207	259.75	8.02	0.005
NR3C2	12	289	24.68	4.63	0.032
STAT5A	11	261	23.68	4.57	0.016
AQP11	14	324	23.49	4.55	0.042
EPB41L3	1	306	227.55	7.83	0.001
BTBD2	20	455	22.62	4.5	0.003
FUT1	1	218	203.98	7.67	0.012
CLN6	16	330	20.81	4.38	0.042
TMA16	1	215	190.89	7.58	0.016
TDRD6	18	351	19.57	4.29	0.041
PPTC7	28	464	16.71	4.06	0.018
SPEF2	2	370	155.08	7.28	0.01
MKNK1-AS1	0	169	1543.4	10.59	0.012
HSPA2	2	371	152.05	7.25	0.03
GUCY1B1	0	152	1386	10.44	0.034
CMC4	2	244	130.85	7.03	0.015
LINC02532	0	141	1288.08	10.33	0.024
ZNF532	28	345	12.32	3.62	0.038
VRK2	74	865	11.69	3.55	0.002
PLOD3	43	482	11.11	3.47	0.024
FOXL2NB	26	292	11.08	3.47	0.025
ZFP64	2	205	106.71	6.74	0.011
EBF1	2	237	105.41	6.72	0.022
LOC101928336	31	329	10.78	3.43	0.043

Down-regulated genes in HCV positive tumor cells

n=159	p-value <0.05	LogFoldChange >2			
Gene id	baseMeanA	baseMeanB	foldChange	og2FoldChang	pval
MAP3K6	95	0	0	inf	0.042
LOC100507634	119	0	0	inf	0.023
PRRX1	137	0	0	inf	0.01
TAF1A-AS1	87	0	0	inf	0.046
CYP26B1	155	0	0	inf	0.004
ARHGAP15	93	0	0	inf	0.033
C2CD6	216	0	0	inf	0.001
SS18L2	139	0	0	inf	0.007
EXOSC7	220	0	0	inf	0
MIR1284	94	0	0	inf	0.036
TACC3	92	0	0	inf	0.035
KIT	171	0	0	inf	0.02

C4orf46	122	0	0	inf	0.014
ESM1	89	0	0	inf	0.049
SHLD3	108	0	0	inf	0.02
C5orf30	153	0	0	inf	0.011
TRIM39	80	0	0	inf	0.05
FAM221A	97	0	0	inf	0.028
LAT2	110	0	0	inf	0.026
SNX16	178	0	0	inf	0.002
TSPYL2	126	0	0	inf	0.009
TBX22	126	0	0	inf	0.047
LINC00894	96	0	0	inf	0.041
HKDC1	139	0	0	inf	0.007
LOC100506606	104	0	0	inf	0.023
PCDH17	134	0	0	inf	0.01
TMEM255B	112	0	0	inf	0.042
AVEN	100	0	0	inf	0.026
PRC1	157	0	0	inf	0.004
FLYWCH1	107	0	0	inf	0.036
LINC01290	184	0	0	inf	0.004
ACSF3	114	0	0	inf	0.015
TLCD1	127	0	0	inf	0.011
ABI3	111	0	0	inf	0.027
PPM1E	168	0	0	inf	0.005
VAV1	99	0	0	inf	0.026
SHKBP1	138	0	0	inf	0.008
PIH1D1	96	0	0	inf	0.035
ZNF649	111	0	0	inf	0.033
ZNF71	89	0	0	inf	0.046
SLPI	87	0	0	inf	0.042
SAYSD1	260	0	0	-10.45	0
LY96	217	0	0	-10.19	0.001
SNORA12	215	0	0	-10.18	0.004
ATG10	261	0	0	-9.71	0
PIK3C2G	155	0	0	-9.7	0.036
MS4A4A	132	0	0	-9.47	0.013
TRAF3IP3	119	0	0	-9.4	0.036
HMGN5	229	0	0	-9.34	0.005
ZNF354A	173	0	0	-9.11	0.025
BICC1	168	0	0	-9.07	0.006
CD3E	143	0	0	-8.84	0.02
PHTF1	168	0	0	-8.83	0.007
ZEB1-AS1	134	0	0	-8.57	0.019
MCM10	118	0	0	-8.31	0.042
ANAPC2	284	1	0	-8.07	0.005
GALK1	139	1	0	-8.04	0.027
CD274	189	1	0	-7.99	0.014
GOLGA6L10	176	1	0	-7.97	0.018
LINC01923	155	1	0	-7.95	0.03
NLGN4Y	180	1	0	-7.93	0.012
HDAC3	163	1	0	-7.78	0.018
SNORA53	145	1	0	-7.68	0.02
GABRP	118	1	0.01	-7.57	0.044
DDIT3	156	1	0.01	-7.54	0.021
MED20	201	1	0.01	-7.49	0.008
APLF	110	1	0.01	-7.47	0.038
TSPYL4	358	2	0.01	-7.25	0
CDK11B	119	1	0.01	-7.13	0.049
ZNF205-AS1	151	1	0.01	-7.03	0.026
LDHB	145	1	0.01	-6.86	0.018
NLRX1	158	1	0.01	-6.76	0.032
CYREN	148	1	0.01	-6.64	0.042

CMSS1	256	3	0.01	-6.52	0.004
CYB5D1	361	4	0.01	-6.51	0.049
SLC36A1	196	2	0.01	-6.49	0.009
PIGC	182	2	0.01	-6.43	0.02
BAD	169	2	0.01	-6.37	0.026
NRP2	368	5	0.01	-6.3	0.001
STEAP2	189	2	0.01	-6.27	0.018
C1orf109	218	3	0.01	-6.26	0.015
ASNSD1	300	4	0.01	-6.22	0.003
LINC01554	170	2	0.01	-6.21	0.039
DHX33	228	3	0.01	-6.08	0.012
CIITA	213	3	0.02	-6.01	0.034
ST7-OT4	171	3	0.02	-5.94	0.041
BRCA1	214	4	0.02	-5.92	0.017
MBP	364	6	0.02	-5.82	0.009
RPAP3	816	15	0.02	-5.78	0
MTMR9	328	6	0.02	-5.76	0.003
C15orf40	212	4	0.02	-5.73	0.017
COLGALT2	172	3	0.02	-5.69	0.042
TSPAN33	320	6	0.02	-5.65	0.008
KIAA0753	198	4	0.02	-5.59	0.022
CDYL2	259	6	0.02	-5.54	0.013
ZNF81	150	3	0.02	-5.51	0.032
HSD17B7	262	6	0.02	-5.37	0.017
CPNE8	189	5	0.03	-5.3	0.03
TBC1D31	221	6	0.03	-5.26	0.041
NUDT16P1	221	6	0.03	-5.16	0.028
AMOT	222	6	0.03	-5.1	0.027
PMVK	216	6	0.03	-5.08	0.031
BBS10	621	19	0.03	-5.06	0
RMDN3	169	5	0.03	-5.05	0.04
AOC3	212	6	0.03	-5.04	0.045
NANP	177	5	0.03	-5.02	0.044
GORAB	360	11	0.03	-5.01	0.006
SLC25A33	166	5	0.03	-4.97	0.034
DNAH12	352	11	0.03	-4.94	0.003
KLHL2	974	34	0.03	-4.84	0
MIPEP	199	7	0.04	-4.79	0.044
SMDT1	192	7	0.04	-4.7	0.04
HDAC1	322	13	0.04	-4.68	0.019
GAN	276	12	0.04	-4.56	0.044
C12orf65	224	10	0.05	-4.45	0.034
CC2D2A	307	16	0.05	-4.3	0.015
HMGXB4	385	20	0.05	-4.28	0.016
SIRT3	274	15	0.05	-4.23	0.043
LIAS	216	12	0.05	-4.22	0.04
GTF2A2	317	17	0.05	-4.22	0.023
WDFY4	268	15	0.06	-4.17	0.027
ACSM1	317	18	0.06	-4.15	0.032
MTX2	360	21	0.06	-4.11	0.007
OARD1	252	15	0.06	-4.1	0.042
HSPB11	270	18	0.07	-3.93	0.03
IMPACT	495	35	0.07	-3.82	0.003
PPCS	483	36	0.07	-3.74	0.007
SNX4	663	53	0.08	-3.65	0.004
SP110	379	36	0.09	-3.41	0.023
FAM206A	244	23	0.1	-3.39	0.041
PPP4C	305	30	0.1	-3.37	0.045
ARHGAP21	512	53	0.1	-3.26	0.008
BTNL9	263	28	0.11	-3.22	0.048
NPEPPS	872	98	0.11	-3.15	0.005

EPB41L2	528	60	0.11	-3.13	0.036
CXCL10	692	88	0.13	-2.97	0.026
PKD2	533	70	0.13	-2.94	0.038
BCL10	423	58	0.14	-2.88	0.032
POLR3GL	472	68	0.14	-2.79	0.028
DAD1	582	88	0.15	-2.72	0.031
SLC25A32	450	70	0.16	-2.68	0.026
PEX3	450	71	0.16	-2.67	0.034
COMMD10	780	125	0.16	-2.65	0.024
GOLT1B	466	78	0.17	-2.58	0.04
ATP6V0E1	782	136	0.17	-2.53	0.015
RPE	538	94	0.17	-2.52	0.043
ATMIN	579	102	0.18	-2.51	0.041
TADA1	513	92	0.18	-2.48	0.027
SNAP29	636	115	0.18	-2.46	0.023
NCK1	713	144	0.2	-2.31	0.024
LAMTOR5	681	138	0.2	-2.3	0.029
FBXL3	825	169	0.2	-2.29	0.005
FBXL17	460	95	0.21	-2.27	0.04
SUMO1	530	112	0.21	-2.24	0.025
DENND5A	587	125	0.21	-2.24	0.024
GALC	391	83	0.21	-2.23	0.044
UTP15	697	155	0.22	-2.17	0.011
AUH	410	94	0.23	-2.13	0.034
VPS37A	730	176	0.24	-2.05	0.047

Up-regulated genes in HCV positive non-tumor cells

n=137 p-value <0.05 LogFoldChange >2

Gene id	baseMeanA	baseMeanB	foldChange	og2FoldChang	pval
MYO19	0	511	Inf	Inf	0,001
MUC13	0	428	Inf	Inf	0,002
ENPP5	0	453	Inf	Inf	0,002
DNPH1	0	425	Inf	Inf	0,003
PRTG	0	412	Inf	Inf	0,004
GPR18	0	371	Inf	Inf	0,004
SLC35B1	0	505	Inf	Inf	0,007
RNF121	0	336	Inf	Inf	0,008
UTP14A	0	357	Inf	Inf	0,009
TTC9	0	397	Inf	Inf	0,011
ABCB6	0	428	Inf	Inf	0,011
RASGRP1	0	307	Inf	Inf	0,012
TAS2R19	0	511	Inf	Inf	0,014
OSER1	0	290	Inf	Inf	0,017
HEPHL1	0	263	Inf	Inf	0,021
CCDC150	0	269	Inf	Inf	0,021
MFSD2A	0	499	Inf	Inf	0,022
RAB39B	0	267	Inf	Inf	0,023
PRSS8	0	266	Inf	Inf	0,024
CGAS	0	344	Inf	Inf	0,024
NDUFB3	0	270	Inf	Inf	0,025
CSTA	0	473	Inf	Inf	0,026
ITPRIP	0	271	Inf	Inf	0,027
HAUS4	0	443	Inf	Inf	0,027
C12orf57	0	428	Inf	Inf	0,028
CYP11A1	0	243	Inf	Inf	0,029
DOCK4-AS1	0	376	Inf	Inf	0,030
CNRIP1	0	321	Inf	Inf	0,032
HEPACAM2	0	251	Inf	Inf	0,032
LOC100131047	0	295	Inf	Inf	0,033

LINC02471	0	281	Inf	Inf	0,034
FANCI	0	277	Inf	Inf	0,035
OR1J2	0	273	Inf	Inf	0,035
SDR42E1	0	265	Inf	Inf	0,035
NCBP2-AS2	0	624	Inf	Inf	0,035
TNFSF8	0	256	Inf	Inf	0,036
GPR174	0	251	Inf	Inf	0,036
FHOD1	0	249	Inf	Inf	0,037
OR51A4	0	235	Inf	Inf	0,037
CBFA2T3	0	349	Inf	Inf	0,038
LRCH2	0	233	Inf	Inf	0,038
LINC00844	0	230	Inf	Inf	0,038
PSKH1	0	227	Inf	Inf	0,038
NOS2P3	0	226	Inf	Inf	0,038
ARL2	0	223	Inf	Inf	0,038
STAT5A	0	263	Inf	Inf	0,039
IL18RAP	0	483	Inf	Inf	0,040
IFNL4	0	220	Inf	Inf	0,040
PGGT1B	0	218	Inf	Inf	0,041
COPG2IT1	0	217	Inf	Inf	0,042
LINC00526	0	429	Inf	Inf	0,042
PFN1P2	0	370	Inf	Inf	0,043
PDZD11	0	401	Inf	Inf	0,044
NLRP14	0	214	Inf	Inf	0,044
GOLGA6L9	0	214	Inf	Inf	0,044
OR4C6	0	389	Inf	Inf	0,045
PLD5	0	385	Inf	Inf	0,045
TPCN2	0	212	Inf	Inf	0,045
NETO2	0	374	Inf	Inf	0,046
HTR3C	0	361	Inf	Inf	0,046
DDX43	0	367	Inf	Inf	0,046
LINC02535	0	359	Inf	Inf	0,046
DDX47	0	337	Inf	Inf	0,047
KLRA1P	0	208	Inf	Inf	0,048
MIS18A	0	235	Inf	Inf	0,049
MTX1	0	314	Inf	Inf	0,050
TRIT1	0	557	1609,26	10,6521783	0,023
TMEM254	0	535	1545,75	10,5940934	0,037
GPC1	0	479	1385,03	10,4357019	0,038
OTUB1	0	380	967,21	9,91768162	0,010
GDF2	0	321	926,53	9,8556961	0,024
NBPF12	0	350	890,38	9,79827653	0,029
TAGAP	0	294	848,45	9,72868648	0,024
SYT1	0	309	787,10	9,62040426	0,049
VWA1	0	299	762,07	9,57377165	0,033
ANO10	0	286	726,70	9,50522025	0,049
SUCLA2	1	755	640,62	9,32332697	0,002
IFT20	2	758	482,37	8,91400796	0,050
TTC19	1	693	469,18	8,87399456	0,000
ME1	1	475	438,13	8,77521552	0,006
MUL1	1	426	361,53	8,49798809	0,009
AGA	1	417	353,78	8,46669493	0,031
GOLT1B	1	442	318,99	8,31735032	0,012
WDHD1	1	360	305,09	8,25308605	0,041
CCND2	2	457	291,01	8,18490495	0,009
METTL14	2	452	287,92	8,16953706	0,011
STIM2	3	822	265,52	8,0526763	0,000
ZNF354B	3	632	229,93	7,84502386	0,018
KLHL42	1	288	201,42	7,65405611	0,046
ATXN1L	3	529	195,79	7,61318702	0,006
JAZF1	3	561	183,82	7,52211458	0,006

HDX	2	354	170,49	7,41356641	0,029
UNC93B1	3	450	157,24	7,29683814	0,015
TRPM8	4	520	142,58	7,15565794	0,007
FBXO34	8	826	100,15	6,64607998	0,001
ZNF766	5	483	99,69	6,63933648	0,028
SFRP1	4	409	95,67	6,57998606	0,034
COIL	5	418	78,60	6,2964074	0,029
CDHR5	7	460	69,93	6,12787304	0,027
STK38	9	574	63,43	5,98700499	0,012
TRIM2	8	461	56,83	5,82866222	0,040
HIF3A	17	727	42,90	5,42279739	0,008
SMAD1	15	607	40,20	5,3291337	0,029
SLC9B2	15	522	35,66	5,15604646	0,046
IL33	15	516	34,66	5,11502287	0,042
GSAP	49	1632	33,46	5,06452081	0,000
MAN2A2	16	517	31,41	4,97315554	0,038
FYB1	26	727	28,39	4,82743017	0,018
MARVELD3	35	967	27,29	4,77035835	0,005
C2orf69	26	683	26,67	4,7370402	0,025
WDR48	27	650	24,37	4,60708664	0,033
ZNF33B	29	640	21,98	4,45797758	0,037
GAREM1	45	913	20,23	4,33825517	0,017
RASSF4	62	1164	18,85	4,2361115	0,008
SNX2	60	991	16,60	4,05341518	0,010
ITGA6	71	1154	16,31	4,02801516	0,008
MMRN1	58	864	14,98	3,90451054	0,034
SIK3	64	818	12,84	3,68275945	0,035
CLOCK	101	1170	11,54	3,52822335	0,011
ATP11A	165	1854	11,25	3,49161695	0,003
FAM160B1	178	1985	11,17	3,48091595	0,003
TMOD3	125	1333	10,67	3,41496455	0,011
RAB5C	105	1012	9,63	3,26681664	0,042
IL7R	85	812	9,58	3,26071938	0,045
CADM1	183	1659	9,05	3,17814903	0,009
LIPA	341	2640	7,73	2,95120516	0,002
MBTPS2	201	1423	7,09	2,82639707	0,037
PPP1CC	198	1271	6,41	2,68095932	0,048
SCOC	254	1503	5,92	2,56609971	0,020
SNX4	327	1893	5,78	2,53201066	0,017
NDUFA5	227	1257	5,55	2,47251363	0,037
CHI3L1	549	2801	5,10	2,35054042	0,007
DAZAP2	503	2283	4,54	2,1826241	0,047
NOTCH2	414	1860	4,49	2,16624672	0,031
MYO6	318	1401	4,41	2,14135539	0,049
ARHGAP5	823	3476	4,22	2,07848702	0,006

Down-regulated genes in HCV positive non-tumor cells

n=65 p-value <0.05 LogFoldChange >2

Gene id	baseMeanA	baseMeanB	foldChange	og2FoldChang	pval
FBXO7	1212	265	0,22	-2,1938995	0,047
HECTD4	1415	184	0,13	-2,9433188	0,013
SPEN	1369	164	0,12	-3,0604314	0,037
CLK1	1463	165	0,11	-3,1454832	0,039
VNN1	1734	188	0,11	-3,206192	0,037
SEC23A	1399	151	0,11	-3,209344	0,033
MSL2	1090	103	0,09	-3,4030123	0,020
VAMP7	1371	114	0,08	-3,5926985	0,039
ZNF217	1040	79	0,08	-3,7109491	0,017
TNKS	860	60	0,07	-3,8319033	0,039

RANBP3L	763	43	0,06	-4,1442161	0,028
CRYBG3	809	33	0,04	-4,6313005	0,020
ABCF1	614	16	0,03	-5,3034608	0,038
CXorf40A	604	13	0,02	-5,5769578	0,027
ABCB10	904	18	0,02	-5,6181014	0,007
DHX38	590	11	0,02	-5,6874768	0,032
PRR11	550	11	0,02	-5,6984878	0,026
AP4E1	629	12	0,02	-5,7333904	0,018
AHSA2P	826	15	0,02	-5,7366375	0,005
TRIM66	534	9	0,02	-5,8963905	0,024
ZNF136	492	8	0,02	-5,9922018	0,024
RPE	653	10	0,02	-6,0562283	0,016
MDC1	516	7	0,01	-6,2227114	0,018
ZNF483	609	8	0,01	-6,2239387	0,010
KIAA0754	401	4	0,01	-6,6231851	0,038
SOX9	367	3	0,01	-6,8190437	0,041
WHAMMP2	382	3	0,01	-6,8745109	0,034
MGC27345	807	6	0,01	-7,0458852	0,023
DENND6A	397	3	0,01	-7,1181953	0,040
GAS5	318	2	0,01	-7,6105441	0,048
PXK	479	2	0,01	-7,6187071	0,022
SMARCD2	403	2	0,01	-7,6287914	0,024
BCL6	504	2	0,00	-7,6819313	0,014
COG6	381	2	0,00	-7,8616653	0,036
TRPS1	441	2	0,00	-8,0826602	0,012
TMED8	338	1	0,00	-8,1099144	0,039
POLR2K	404	1	0,00	-8,3698396	0,018
NYNRIN	559	2	0,00	-8,4249826	0,043
HCCS	434	1	0,00	-8,4659793	0,018
CYP1A1	336	1	0,00	-8,69251	0,029
RBX1	325	0	0,00	-9,6327698	0,034
AP5B1	344	0	0,00	-9,7163261	0,027
GALK2	424	0	0,00	-10,017367	0,010
ANKRD46	606	0	0,00	"-inf"	0,001
UBE2E3	453	0	0,00	"-inf"	0,004
SLC5A9	361	0	0,00	"-inf"	0,013
KDM8	336	0	0,00	"-inf"	0,018
ISG20	322	0	0,00	"-inf"	0,021
TPX2	323	0	0,00	"-inf"	0,021
ATF1	312	0	0,00	"-inf"	0,024
LINC02338	311	0	0,00	"-inf"	0,024
ZNF441	366	0	0,00	"-inf"	0,026
SIGLEC9	304	0	0,00	"-inf"	0,027
PARG	303	0	0,00	"-inf"	0,027
CHMP6	297	0	0,00	"-inf"	0,029
STAT4	288	0	0,00	"-inf"	0,032
GET4	288	0	0,00	"-inf"	0,033
TIMM9	288	0	0,00	"-inf"	0,033
NDUFAF8	283	0	0,00	"-inf"	0,034
FAM174A	278	0	0,00	"-inf"	0,037
MOCOS	276	0	0,00	"-inf"	0,039
GPR37	295	0	0,00	"-inf"	0,044
GARNL3	265	0	0,00	"-inf"	0,045
INPP5F	257	0	0,00	"-inf"	0,049

Supplementary Table S3- Gene Ontology Analysis (DAVID)

Non Tumor HCV+ vs HCV-					
Category	Term	Count	%	P-Value	Fold
GOTERM_CC_DIRECT	Golgi membrane	12	9	1,50E-03	3,1
GOTERM_CC_DIRECT	integral component of membrane	45	33,8	1,80E-02	1,3
GOTERM_BP_DIRECT	centrosome organization	3	2,3	1,90E-02	14,1
GOTERM_MF_DIRECT	leptin receptor binding	2	1,5	2,00E-02	100,5
GOTERM_BP_DIRECT	skin development	3	2,3	2,00E-02	13,7
GOTERM_CC_DIRECT	intracellular	16	12	2,30E-02	1,9
GOTERM_BP_DIRECT	ion transmembrane transport	5	3,8	4,50E-02	3,7
GOTERM_CC_DIRECT	MLL5-L complex	2	1,5	5,00E-02	38,6
GOTERM_MF_DIRECT	transferrin receptor binding	2	1,5	5,10E-02	37,7
GOTERM_BP_DIRECT	cartilage development	3	2,3	5,40E-02	7,9
GOTERM_CC_DIRECT	endosome	5	3,8	5,70E-02	3,4
GOTERM_CC_DIRECT	cell-cell adherens junction	6	4,5	5,70E-02	2,9
GOTERM_CC_DIRECT	lysosome	5	3,8	5,80E-02	3,4
GOTERM_MF_DIRECT	identical protein binding	10	7,5	5,80E-02	2
GOTERM_BP_DIRECT	protein deglycosylation	2	1,5	6,20E-02	31,1
GOTERM_BP_DIRECT	positive regulation of T cell differentiation in thymus	2	1,5	6,20E-02	31,1
GOTERM_BP_DIRECT	succinate metabolic process	2	1,5	6,20E-02	31,1
GOTERM_CC_DIRECT	mitochondrial outer membrane	4	3	7,10E-02	4,1
GOTERM_BP_DIRECT	cell division	6	4,5	7,30E-02	2,7
GOTERM_BP_DIRECT	cellular response to interleukin-1	3	2,3	7,50E-02	6,6
GOTERM_BP_DIRECT	homeostasis of number of cells	2	1,5	8,00E-02	23,9
GOTERM_CC_DIRECT	plasma membrane	34	25,6	9,20E-02	1,3
GOTERM_BP_DIRECT	positive regulation of cartilage development	2	1,5	9,70E-02	19,4

Tumors HCV+ vs HCV-					
Category	Term	Count	%	P-Value	Fold
GOTERM_MF_DIRECT	metal ion binding	19	20,4	1,10E-03	2,2
GOTERM_MF_DIRECT	DNA binding	15	16,1	6,20E-03	2,2
GOTERM_BP_DIRECT	transcription, DNA-templated	16	17,2	1,40E-02	1,9
GOTERM_BP_DIRECT	regulation of cell shape	4	4,3	2,10E-02	6,8
GOTERM_BP_DIRECT	regulation of transcription, DNA-templated	13	14	2,10E-02	2
GOTERM_MF_DIRECT	transcription factor activity, sequence-specific DNA bin	9	9,7	3,90E-02	2,3
GOTERM_BP_DIRECT	endothelial cell morphogenesis	2	2,2	4,50E-02	43
GOTERM_BP_DIRECT	regulation of synaptic transmission, GABAergic	2	2,2	4,90E-02	39,4
GOTERM_BP_DIRECT	fatty acid oxidation	2	2,2	5,70E-02	33,8
GOTERM_BP_DIRECT	lipoprotein transport	2	2,2	6,10E-02	31,5
GOTERM_BP_DIRECT	glucose homeostasis	3	3,2	6,60E-02	7
GOTERM_BP_DIRECT	male meiosis	2	2,2	8,80E-02	21,5
GOTERM_MF_DIRECT	metalloaminopeptidase activity	2	2,2	8,90E-02	21,3
GOTERM_BP_DIRECT	negative regulation of cell growth	3	3,2	9,10E-02	5,9
